# Factorials of real negative and imaginary numbers - A new perspective

**DOI:** 10.1186/2193-1801-3-658

**Published:** 2014-11-06

**Authors:** Ashwani K Thukral

**Affiliations:** Department of Botanical & Environmental Sciences, Guru Nanak Dev University, Amritsar, 143005 India

**Keywords:** Factorials of negative numbers, Factorials of imaginary numbers, Pi function, Fractional factorials, Multifactorials, Gamma function, Beta function

## Abstract

Presently, factorials of real negative numbers and imaginary numbers, except for zero and negative integers are interpolated using the Euler’s gamma function. In the present paper, the concept of factorials has been generalised as applicable to real and imaginary numbers, and multifactorials. New functions based on Euler’s factorial function have been proposed for the factorials of real negative and imaginary numbers. As per the present concept, the factorials of real negative numbers, are complex numbers. The factorials of real negative integers have their imaginary part equal to zero, thus are real numbers. Similarly, the factorials of imaginary numbers are complex numbers. The moduli of the complex factorials of real negative numbers, and imaginary numbers are equal to their respective real positive number factorials. Fractional factorials and multifactorials have been defined in a new perspective. The proposed concept has also been extended to Euler’s gamma function for real negative numbers and imaginary numbers, and beta function.

## Background

The factorial of a positive integer, *n*, is defined as,


The factorials of positive integers follow the recurrence relation,


The factorials of negative integers cannot be computed, since for *n* = 0, the recurrence relation,


involves a division by zero. Research on the interpolation of factorials started with correspondence among Leonhard Euler, Daniel Bernoulli and Christian Goldbach in the year 1729 (Refer to the correspondence reproduced by Dartmouth College 2014; and Luschny 2014a). Bernoulli in the year 1729 gave an interpolating function of factorials as an infinite product (Gronau 2003). Euler in the year 1730 proved that the integral,
1

gives the factorial of *x* for all real positive numbers (Srinivasan 2007). Euler’s factorial function, also known as the Pi function, Π(*x*), follows the recurrence relation for all positive real numbers.


In 1768, Euler defined the gamma function, Γ(z), and extended the concept of factorials to all real negative numbers, except zero and negative integers. Γ(z), is an extension of the Pi function, with its argument shifted down by 1. Also known as the Euler’s integral of the second kind (Gautschi 2008), it is a convergent improper integral defined as follows:
2

**Figure 1:**
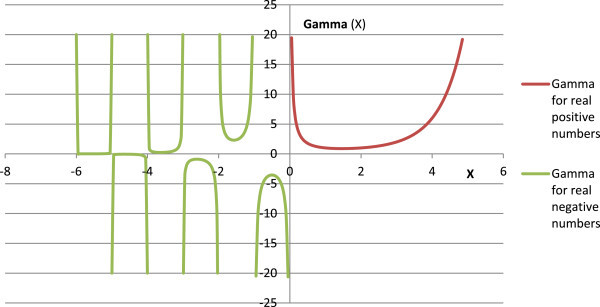
**A plot of Euler’s gamma function.**

The Euler’s gamma function is related to the Pi function as follows:


The notation ‘!’ for the factorial function was introduced by C. Kramp in the year 1808 (Wolfram Research 2014a,[b]). Legendre in 1808 gave the notation ‘Γ’ to the Euler’s gamma function (Gronau 2003). Gauss introduced the notation


which was subsequently abandoned and replaced with Legendre’s notation (Weistein 2014b). C.F. Gauss made important contributions to derive several important properties of the gamma function (Srinivasan 2007). Anglani and Barlie (2007) gave the additive representation of factorials. The gamma function is extended to all complex numbers, with a real part >0, except for at zero and negative integers. Figure 1 gives the curve for gamma function (Eqn. 2). At negative integers, the gamma function has simple poles, making it a meromorphic function (Figure 1).

It may be seen that the gamma function defined for *x* > -1 has been used to interpolate the factorials on the real negative axis. Similarly, the factorials of complex numbers are calculated from the gamma function (Wolfram 2014b), such as,


and


where *x* is a real number. Among the other well defined functions for the factorials of real negative numbers are, Hadamard’s gamma function (Davis 1959) and Luschny’s factorial function (Luschny 2014b), both of which are continuous and positive at all real numbers. Roman (1992) defined the factorials of negative integers as under:


where Roman ⌊*n*⌉ is defined as *n*, for *n* ≠ 0, and 1 for *n* =0. Roman factorials of first few negative integers (Loeb 1995) are given in Table 1.

**Table 1:** **Roman factorials**

***n***	Roman factorial ⌊ ***n***⌉!
0	1
-1	1
-2	-1
-3	1/2
-4	-1/6
-5	1/24
-6	-1/120

The other notable contributors to the field of factorials are J. Stirling, F.W. Newman, B. Riemann, H. Hankel, O. Holder, H. Bohr and J. Mollerup, and others (Wolfram Research 2014b). Dutka (1991) gave an account of the early history of the factorial function. Bhargava (2000) gave an expository account of the factorials, gave several new results and posed certain problems on factorials. Ibrahim (2013) defined the factorial of negative integer *n* as the product of first *n* negative integers. There are some other factorial like products and functions, such as, primordial, double factorial, multifactorials, superfactorial, hyperfactorials etc. (Wikipedia 2014a,[b]; Weistein 2014a).

It is seen that till now the definition of the factorials of real negative numbers is sought from the extrapolation of gamma and other functions. In the present paper, the Eularian concept of factorials has been revisited, and new functions based on Euler’s factorial function (Eqn. 1) have been defined for the factorials of real negative numbers and imaginary numbers.

### 1. Factorials of real negative numbers

Let *a*_n_ be a sequence of positive integers, *a*_n=1,2,3,…,*n*._ Therefore,

*n*! = 1.2.3…*n*.

Multiplying each integer on the right hand side of *a*_n_ with a constant, *c* ≠ 0, termed here as factorial constant, we get
3

Putting *c* = -1 gives,
4

The expression (-1)^*n*^*n* ! on the left hand side of Eqn. (4), gives the product of first *n* consecutive negative integers and may be termed as the factorials of negative integers (Table 2). For convenience Eqn. (4) may be presented as (-*n*)!.

**Table 2:** **Factorials of some integers as per present concept**

***n***	***n***!	-***n***	(-***n***)! = [(-1) ^***n***^ ***n***!]
1	1	-1	-1
2	2	-2	2
3	6	-3	-6
4	24	-4	24
5	120	-5	-120

In the present communication, a new function obtained from the Euler’s factorial function (Eqn. 1) has been proposed to interpolate the factorials of real negative numbers as given below:
5

where *z* is a real positive number, and *c* is a factorial constant not equal to zero, and Π(*c,z*) is modified Euler’s factorial function. For *c* =1, the factorial for real positive numbers is defined as per Euler’s factorial function (Eqn. 1). For *c* = -1, factorials of real negative numbers as described by Eqn. (5) can be interpolated as follows:


Or,
6

where Π(-1,*z*) is the factorial of the negative real number (-*z*) as per the present concept. For the real negative axis, Eqn. (6) may be written as
7

**Figure 2:**
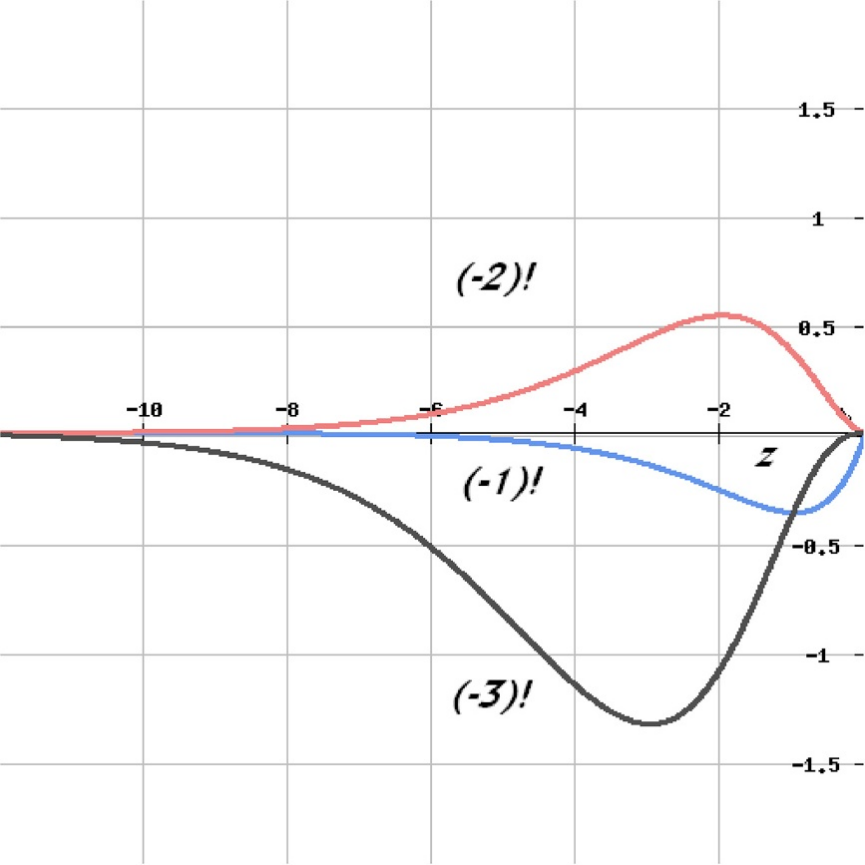
**Curves for the integral functions of factorials of some negative integers on the real negative axis.**

Analogous to factorials of real positive numbers, the factorials of real negative numbers, Π(-1,z) may be given by the notation(-*z*)!. Figure 2 gives the curves for the integral functions of factorials of real negative integers, (-1), (-2), (-3), on the real negative axis. The area between a curve and the X-axis gives the factorial of that number.

The factorials of some real negative numbers as given by Eq. (6) are given in Table 3. The factorial of a real negative number is a complex number, represented as


where *x* is the real part and *y* is imaginary. The factorial of 0 is 1. At real negative integers the imaginary part is zero and the real part has alternating – and + signs, with the factorial of (-1) being (-1). The most important property that justifies the present concept is that the moduli of the complex factorials of real negative numbers are equal to the factorials of real positive numbers.

It is also seen from Table 3, that for real negative numbers, at half fractions the real part is zero, at ¼ fractions, the real and imaginary parts are equal, and at ¾ fractions the real and imaginary parts are equal in magnitude but opposite in +/- sign.

**Table 3:** **Complex factorials of some real negative numbers**

	Real	Imaginary	Modulus	Im/Re
*z*	Complex factorial of (-*z*)			
0	1	0	1	0
0.25	0.640	0.640*i*	0.906	1
0.5	0	0.886*i*	0.886	Comp Inf
0.75	-0.649	0.649*i*	0.919	-1
1	-1	0	1	0
1.25	-0.801	-0.801*i*	1.133	1
1.5	0	-1.329*i*	1.329	Comp Inf
1.75	1.137	-1.137*i*	1.608	-1
2	2	0	2	0
2.25	1.802	1.802*i*	2.549	1
2.5	0	3.323*i*	3.323	Comp Inf
2.75	-3.127	3.127*i*	4.422	-1
3	-6	0	6	0

Factorials of real negative numbers as proposed above follow recurrence relations:


### 1.1 Factorials of half fractions of real negative numbers

Let Z = *n* + 0.5, *n* ≥ 0, then


Thus, the real part of the complex factorials of negative real numbers will be zero at negative half integers. At *z* = -0.5


The factorials of -0.25 and -0.75 will be


**Figure 3:**
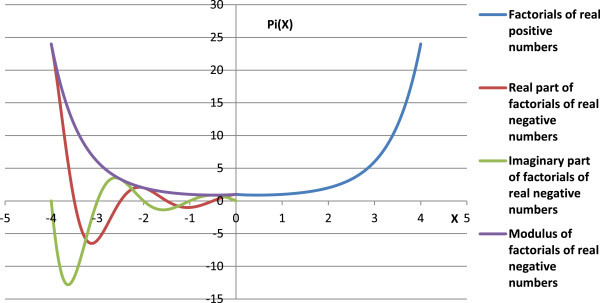
**Factorials of real numbers using Euler’s PI function (right) and the present** **(-1,** ***z*** **)** **function (left).**

**Figure 4:**
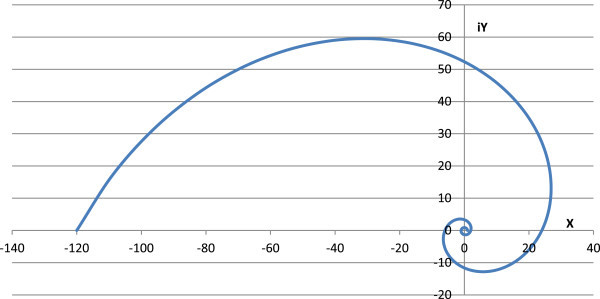
**Polar line graph between real (X-axis) and imaginary (Y-axis) parts of factorials of negative real numbers using** **(-1,** ***z*** **)** **.**

**Figure 5:**
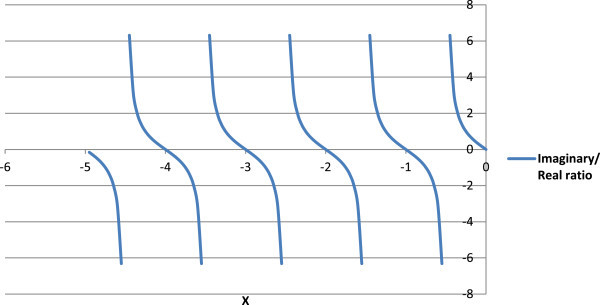
**Ratio of imaginary to real part of factorials of negative numbers (Y-axis) using** **(-1,** ***z*** **)** **function.**

Figure 3 shows the Euler’s factorials Π(*z*) of real positive numbers (Eqn. 1) and factorials of real negative numbers as per proposed Π(-1,*z*) function (Eqn. 6). Figure 4 gives the polar line graph between real and imaginary parts of complex factorials of negative real numbers, and Figure 5 describes the ratio of imaginary to real parts of complex factorials of real negative numbers. The polar graph of real X-axis *vs*. imaginary Y-axis for Π(-1,*z*) function, and tan θ, () are shown in Figure 5.

### 1.2 Exponential function

Exponential function can be represented in terms of factorials (Oldham et al. 2009):


Substituting the positive factorials with negative factorials we get,


## 2. Factorials of imaginary numbers

Similar to the factorials of real positive and real negative integers as defined in Eqn. (3), we may define the factorials of imaginary positive integers as
8

For convenience (*i*)^*n*^*n* ! may be written as (*in*)! In the integral form, Eqn. (8) may be written as,
9

Similarly, factorials of imaginary negative integers may be defined as
10

The integral form of Eqn. (10) may be defined as
11

Complex factorials of imaginary numbers will be related to the factorials of the respective real numbers as follows:
1213

**Figure 6:**
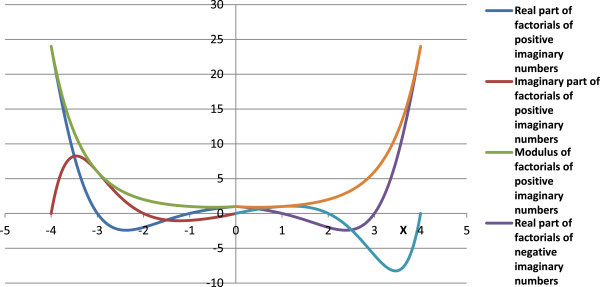
**Graph of complex factorials of imaginary numbers.**

**Figure 7:**
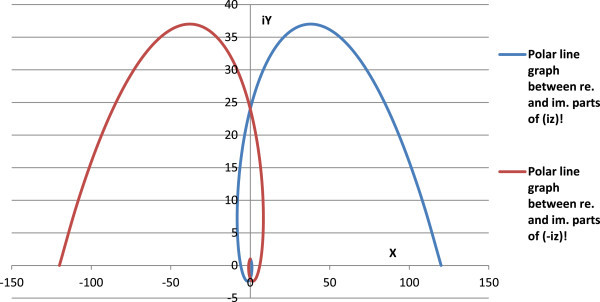
**Polar line graph between real (X-axis) and imaginary (Y-axis) parts of complex factorials of imaginary numbers.**

**Figure 8:**
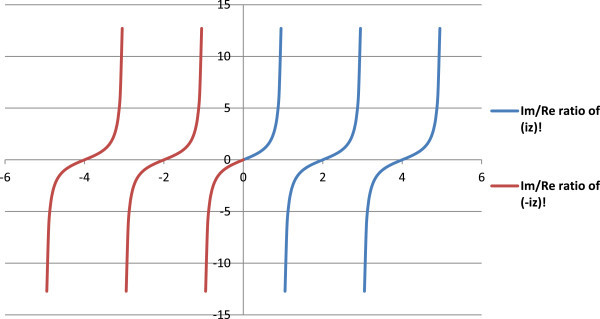
**Ratio of imaginary to real parts of complex factorials of imaginary numbers (Y-axis) as per the present concept.**

Complex factorials of some imaginary numbers as calculated from Eqn. (12, 13) are given in Table 4, and Figures 6, 7 and 8. The modulus of the complex factorial of an imaginary number (*iz*) or (-*iz*) is equal to the factorial of the respective real number (*z*).

**Table 4:** **Complex factorials of some imaginary numbers**

*z*	Complex factorial of (*iz* )			
	Real	Imaginary	Modulus	Im/Re
0	1	0	1	0
0.25	0.837	0.346*i*	0.906	2.414
0.5	0.626	0.626*i*	0.886	1
0.75	0.351	0.849*i*	0.919	2.414
1	0	*i*	1	Comp Inf
1.25	-0.433	1.046*i*	1.133	-0.414
1.5	-0.939	0.939*i*	1.329	-1
1.75	-1.485	0.615*i*	1.608	-2.414
2	-2	0	2	0
2.25	-2.355	-0.975*i*	2.549	2.414
2.5	-2.349	-2.349*i*	3.323	1
2.75	-1.692	-4.086*i*	4.422	0.414
3	0	-6*i*	6	Comp Inf
***z***	**Complex factorial of (-** ***iz*** **)**			
0	1	0	1	0
0.25	0.837	-0.346*i*	0.906	-0.414
0.5	0.626	-0.626*i*	0.886	-1
0.75	0.351	-0.849*i*	0.919	-2.414
1	0	-*i*	1	Comp Inf
1.25	-0.433	-1.046*i*	1.133	2.414
1.5	-0.939	-0.939*i*	1.329	1
1.75	-1.485	-0.615*i*	1.608	0.414
2	-2	0	2	0
2.25	-2.355	0.975*i*	2.549	-2.414
2.5	-2.349	2.349*i*	3.323	-1
2.75	-1.692	4.086*i*	4.422	-0.414
3	0	6*i*	6	Comp Inf

The factorial of an imaginary number (*iz*)! or (-*iz*)! may be represented as a product of the coefficient (*i*^*z*^) or (-*i*)^*z*^ and *z*! (Eq. 12, 13). The coefficients of factorials of imaginary integers follow a periodicity of four (Table 5).

**Table 5:** **Periodicity of factorials of imaginary numbers**

Z	(***iz***) !	Coeff.	(-***iz***) !	Coeff.
1	*Π*(*i*, 1) = *iΠ*(1) = *i*	*i*	*Π*(-*i*, 1) = - *iΠ*(1) = - *i*	-*i*
2	*Π*(*i*, 2) = - *Π*(2) = - 2	-1	*Π*(-*i*, 2) = - *Π*(2) = - 2	-1
3	*Π*(*i*, 3) = - *iΠ*(3) = - 6*i*	-*i*	*Π*(-*i*, 3) = *iΠ*(3) = 6*i*	*i*
4	*Π*(*i*, 4) = *Π*(4) = 24	1	*Π*(-*i*, 4) = *Π*(4) = 24	1

Factorials of imaginary numbers follow recurrence relations:


### 2.1 Factorials of half fractions of imaginary numbers

Let *z* = *n* + 0.5, *n* ≥ 0, then


Similarly, for imaginary negative numbers,


Factorials of 0.5*i* and -0.5*i* are given as


## 3. Multifactorials and fractional factorials

Let *c*_*n*_ be a sequence defined by Eqn. (3).


where *c* ≠ 0 is a constant multiplier of the terms of the sequence, called here as the factorial constant, and * represents multiplication. The product of the terms of the sequence may be called factorial of the sequence and represented as Π(*c*,*n*). It is given as,


If *c* is a fraction, the product may be called fractional factorial. For example, if *c* =0.5,


If *c* is an integer >1, e.g., 2, 3 etc., we get multifactorials.


For example, if *c* =2, and n =4, we get the double factorial of c*n =8,


Fractional factorials and multifactorials can be interpolated using Eqn. (5), where *c* ≠ 0 is a fraction of a real or imaginary number.


The value of fractional factorials and multifactorials at zero is 1. Fractional factorials and multifactorials of some real and imaginary numbers are given in Table 6.

**Table 6:** **Fractional factorials and multifactorials**

Fractional factorials and multifactorials of real positive numbers					
		Fractional factorials		Multifactorials	
*z*	*z*!	(0.5*z*)!	(1.5*z*)!	(2*z*)!!	(3*z*)!!!
0	1	1	1	1	1
0.5	0.886	0.626	1.085	1.253	1.534
1	1	0.5	1.5	2	3
1.5	1.329	0.469	2.442	3.759	6.907
2	2	0.5	4.5	8	18
2.5	3.323	0.587	9.158	18.799	51.805
3	6	0.75	20.25	48	162
**Fractional factorials and multifactorials of real negative numbers**					
		**Fractional factorials**		**Multifactorials**	
*z*	(-*z*)!	(-0.5)*z*!	(-1.5*z*)!	(-2*z*)!!	(-3*z*)!!!
0	1	1	1	1	1
0.5	0.886*i*	0.626*i*	1.085	1.253*i*	1.534*i*
1	-1	-0.5	-1.5	-2	-3
1.5	-0.139*i*	-0.469*i*	-2.442	-3.759*i*	-6.907*i*
2	2	0.5	4.5	8	18
2.5	3.323*i*	0.587	9.157	18.799*i*	51.805*i*
3	-6	-0.75	-20.25	-48	-162
**Fractional factorials and multifactorials of imaginary positive numbers**					
		**Fractional factorials**		**Multifactorials**	
*z*	(*iz*!)	(0.5*iz*)!	(1.5*iz*)!	(2*iz*)!!	(3*iz*)!!!
0	1	1	1	1	1
0.5	0.626 + 0.626*i*	0.443 + 0.443*i*	0.767 + 0.767*i*	0.886 + 0.886*i*	1.085 + 1.085*i*
1	*i*	0.5*i*	1.5*i*	2*i*	3*i*
1.5	-0.939 + 0.939*i*	-0.332 + 0.332*i*	-1.726 + 1.726*i*	-2.658 + 2.658*i*	-4.862 + 4.862*i*
2	-2	-0.5	-4.5	-8	-18
2.5	-2.349-2.349*i*	-0.415-0.415*i*	-6.475-6.475*i*	-13.29-13.29*i*	36.6-36.6*i*
3	6*i*	-0.75*i*	-20.25*i*	-48*i*	-162*i*
**Fractional factorials and multifactorials of imaginary negative numbers**					
		**Fractional factorials**		**Multifactorials**	
*z*	(-*iz*)!	(-0.5*iz*)!	(-1.5*iz*)!	(-2*iz*)!!	(-3*iz*)!!!
0	1	1	1	1	1
0.5	0.626-0.626*i*	0.443-0.443*i*	0.767-0.767*i*	0.886-0.886*i*	1.085-1.085*i*
1	*-i*	-0.5i	-1.5*i*	-2*i*	-3*i*
1.5	-0.939-0.939*i*	-0.332-0.332*i*	-1.726-1.726*i*	-2.658-2.658*i*	-4.862-4.862*i*
2	-2	-0.5	-4.5	-8	-18
2.5	-2.349 + 2.349*i*	-0.415 + 0.415*i*	-6.475 + 6.475*i*	-13.29 + 13.29*i*	-36.6 + 36.6*i*
3	6*i*	0.75*i*	20.25*i*	48*i*	162*i*

The modulus of the fractional factorials and multifactorials of real and imaginary numbers as proposed above follow recurrence relations:


## 4. Gamma function

As per the present concept, the Euler’s gamma function (Eq. 2) may be modified as,


For real negative numbers the gamma will be,
14

**Figure 9:**
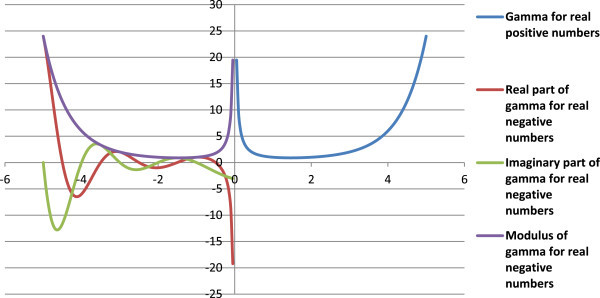
**A plot of the gamma function for real numbers as per the proposed concept.**

For the negative Z-axis, gamma will be given as,


The recurrence relation of negative gamma is:


Negative gamma will be related to negative factorial function as follows:


Gamma values of real negative numbers are given in Table 7. Figure 9 represents the gamma of factorials of real negative numbers as per the present concept (Eqn. 14).

**Table 7:** **Complex gamma of real negative and imaginary numbers**

Complex gamma of (-***z***)				
***z***	Real	Imaginary	Modulus	Im/Re
0.05	-19.230	-3.045*i*	19.470	0.158
0.25	-2.563	-2.563*i*	3.625	1
0.5	0	-1.772*i*	1.772	Comp Inf
0.75	0.866	-0.866*i*	1.225	-1
1	1	0	1	0
**Complex gamma of (** ***iz*** **)**				
***z***	**Real**	**Imaginary**	**Modulus**	**Im/Re**
0.05	1.527	-19.410*i*	19.470	-12.706
0.25	1.387	-3.349*i*	3.625	-2.414
0.5	1.253	-1.253*i*	1.772	-1
0.75	1.132	-0.468*i*	1.225	-0.414
1	1	0	1	0
**Complex gamma of (-** ***iz*** **)**				
***z***	**Real**	**Imaginary**	**Modulus**	**Im/Re**
0.05	19.410	1.527*i*	19.470	12.706
0.25	3.349	1.387*i*	3.625	2.414
0.5	1.253	1.253*i*	1.772	1
0.75	0.468	1.132*i*	1.225	0.414
1	0	*i*	1	Comp Inf

Similar to the gamma of real negative numbers (Eqn. 14), gamma of imaginary positive numbers will be
15

For imaginary negative numbers, gamma will be
16

**Figure 10:**
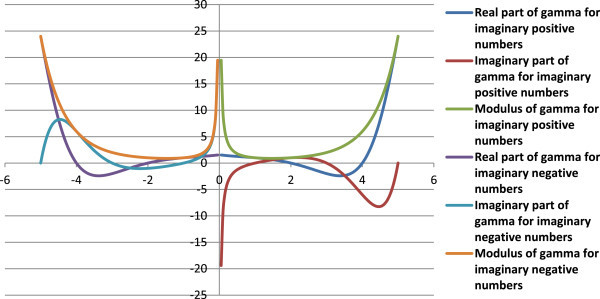
**A plot of the gamma function for imaginary numbers as per the proposed concept.**

**Figure 11:**
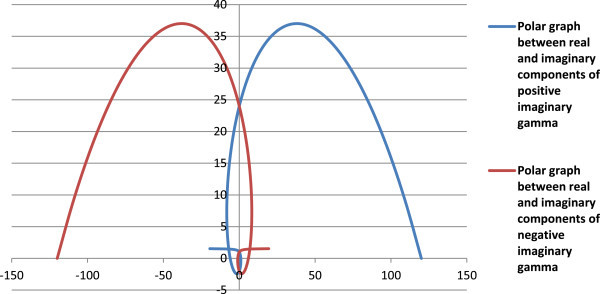
**Polar graph for real (X-axis)** ***vs*** **. imaginary (Y-axis) components of gamma of imaginary numbers as per the present hypothesis.**

Figures 10 and 11 represent the gamma for imaginary numbers.

## 5. Beta function

The present concept of negative factorials can also be extended to beta function, also known as Euler’s factorial of first kind. Beta may be defined as (Culham 2014; Weistein 2014c; Wikipedia 2014c):


The gamma of negative numbers as per the present concept will be,


Therefore, the beta function of negative numbers, as per the present concept may therefore be defined as


**Figure 12:**
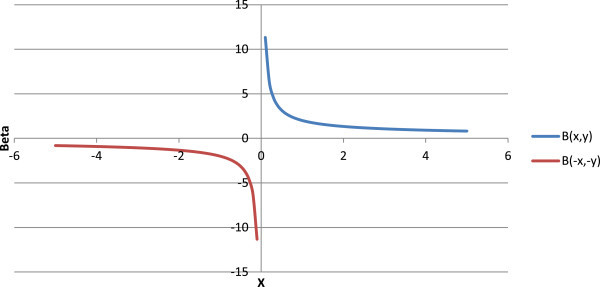
**Graph for B(** ***x*** **,0.5) and B(-** ***x*** **,-0.5) as per the present concept.**

The graph of beta function is given in Figure 12.

It is seen from the historical account that the Euler’s contributions to logarithms and gamma function have revolutionized developments in science and technology (Lefort 2002; Lexa 2013). Factorial function was first defined for the positive real axis. Later its argument was shifted down by 1, and the factorial function was extended to negative real axis and imaginary numbers. Recently, the author (Thukral and Parkash 2014; Thukral 2014) gave a new concept on the logarithms of real negative and imaginary numbers. Earlier the logarithms of real negative numbers were defined on the basis of hyperbola defined for the first quadrant and extended to the negative real axis, but the author defined the logarithms for the real negative axis on the basis of hyperbola located in the third quadrant. Similarly, the author in this paper has defined the factorial function for the real negative axis. The factorials of real and imaginary numbers thus defined show uniformity in magnitude and satisfy the basic factorial equation (*c*)^*n*^*n* ! = *c*(*c*2)(*c*3) … (*cn*). Another lacuna in the existing Eularian concept of factorials is that although the factorials of negative integers are not defined, the double factorial of any negative odd integer may be defined, e.g., (-1)!! =1, (-3)!! = -1, (-5)!! =1/3 etc. (Wikipedia 2014b). Another strange behaviour of double factorials is that as an empty product, 0!! =1 but for non-negative even integer values, . The present concept will remove anomalies in factorials and double factorials. The present concept generalizes factorials as applicable to real and imaginary numbers, and fractional and mutifactorials.

## Conclusions

The present paper examines the Eularian concept of factorials from basic principles and gives a new concept, based on the Eularian concept for factorials of real negative and imaginary numbers. The factorials of positive and negative integers, and positive and negative imaginary number integers (*z*), may be defined as *Π*(*c*, *z*) = *c*^*z*^*z* ! = *c*(*c*2)(*c*3) … (*cn*), where *c* is a constant (+1, -1, +*i* or –*i*), and *z* >0. The factorials can be interpolated using the Euler’s modified integral equation,  for real and imaginary numbers. The factorials for real negative numbers may be defined by the integral equation, . The factorials of negative real numbers are complex numbers. At negative integers the imaginary part of complex factorials is zero, and the factorials for -1, -2, -3, -4 are -1, 2, -6, 24 respectively. Similarly, the factorials of imaginary numbers are complex numbers. The moduli of negative real number factorials and imaginary number factorials are equal to the factorials of respective real positive numbers. The present paper also provides a general definition of fractional factorials and multifactorials. The factorials follow recurrence relations. Similarly, the Euler’s gamma function has been redefined for negative real and imaginary numbers in a new perspective. Beta function on the real negative axis has also been redefined in the context of new concept. The present concept on factorials will be an improvement in the Euler’s factorial and gamma functions.

### Software used

Following softwares were used in this paper:

1. Wolfram Alpha Examples: Complex Numbers (http://www.wolframalpha.com/examples/ComplexNumbers.html)
2. Draw Function Graphs – Recheronline. (http://rechneronline.de/function-graphs/)
3. Definite integral calculator from Wolfram Alpha Widgets by Evan added in 2010 (http://www.wolframalpha.com/input/?i=definite%20integral%20calculator)
4. Integral calculator from Wolfram Mathematica Online integrator (http://integrals.wolfram.com/index.jsp)
5. Gamma Function Evaluator – The Wolfram Functions site http://functions.wolfram.com/webMathematica/FunctionEvaluation.jsp?name=Gamma
6. Function calculator by XIAO gang 2012 (http://wims.unice.fr/wims/en_tool~analysis~function.en.html)
7. Microsoft Excel.
